# *N-*Oleoylglycine and *N*-Oleoylalanine Do Not Modify Tolerance to Nociception, Hyperthermia, and Suppression of Activity Produced by Morphine

**DOI:** 10.3389/fnsyn.2021.620145

**Published:** 2021-03-09

**Authors:** Erin M. Rock, Cheryl L. Limebeer, Megan T. Sullivan, Marieka V. DeVuono, Aron H. Lichtman, Vincenzo Di Marzo, Raphael Mechoulam, Linda A. Parker

**Affiliations:** ^1^Department of Psychology and Collaborative Neuroscience Program, University of Guelph, Guelph, ON, Canada; ^2^Department of Pharmacology and Toxicology, Virginia Commonwealth University, Richmond, VA, United States; ^3^Endocannabinoid Research Group, Institute of Biomolecular Chemistry, Consiglio Nazionale delle Richerche, Naples, Italy; ^4^Canada Excellence Research Chair on the Gut Microbiome/Endocannabinoidome Axis in Metabolic Health, Faculty of Medicine and Faculty of Agriculture and Food Science, CRIYUCPQ, INAF and Centre NUTRISS, Université Laval, Quebec City, QC, Canada; ^5^Medical Faculty, Institute for Drug Research, Hebrew University, Jerusalem, Israel

**Keywords:** *N-*oleoylglycine, *N*-oleoylalanine, morphine, tolerance, analgesia

## Abstract

The endogenous amide *N*-Oleoylglycine (OlGly) and its analog *N*-Oleoylalanine (OlAla), have been shown to interfere with the affective and somatic responses to acute naloxone-precipitated MWD in male rats. Here we evaluated the potential of a single dose (5 mg/kg, ip) which alleviates withdrawal of these endogenous fatty acid amides to modify tolerance to anti-nociception, hyperthermia, and suppression of locomotion produced by morphine in male Sprague-Dawley rats. Although rats did develop tolerance to the hypolocomotor and analgesic effects of morphine, they did not develop tolerance to the hyperthermic effects of this substance. Administration of neither OlGly nor OlAla interfered with the establishment of morphine tolerance, nor did they modify behavioral responses elicited by morphine on any trial. These results suggest that the effects of OlGly and OlAla on opiate dependence may be limited to naloxone-precipitated withdrawal effects.

## Introduction

*N-*Oleoylglycine (OlGly) is an endogenous fatty acid amide that is structurally similar to *N-*aceylethanolamines, including the endocannabinoid, anandamide (AEA), as well as the endogenous peroxisome proliferator-activated receptor alpha (PPARα) agonists, *N-*oleoylethanolamide (OEA) and *N-*palmitoylethanolamide (PEA). Endogenous OlGly is released in the insular cortex of mice experiencing traumatic brain injury (Donvito et al., 2019). Since the insular cortex is a site critical for nicotine addiction (Naqvi et al., 2007, 2014; Forget et al., 2010), the laboratory of Raphael Mechoulam synthesized OlGly for evaluation of the potential of this fatty acid to mitigate nicotine addiction. Indeed, synthetic OlGly interfered with nicotine reward and dependence in mice. OlGly did not bind with cannabinoid 1 (CB_1_) or cannabinoid 2 (CB_2_) receptors *in vitro* and did not produce the typical CB_1_ receptor tetrad (antinociception, hypothermia, catalepsy, and hypomobility) of behaviors in mice. OlGly was shown to bind with PPARα and to inhibit fatty acid amide hydrolase (FAAH) *in vitro.* Indeed, the interference with nicotine reward by OlGly was reversed by a PPARα antagonist, a finding consistent with previous reports of PPARα agonists interfering with nicotine reward (Mascia et al., 2011; Justinova et al., 2015; Jackson et al., 2017). In contrast, a CB_1_ receptor antagonist did not reverse OlGly interference with nicotine reward (Donvito et al., 2019).

Not only has OlGly been shown to interfere with nicotine dependence, but also with the aversive (Petrie et al., 2019) and somatic (Rock et al., 2020) withdrawal produced by acute naloxone-precipitated morphine withdrawal (MWD), at a dose of 1 or 5 mg/kg, intraperitoneal (ip), but not 20 mg/kg, ip, in rats. On the other hand, OlGly neither modified a morphine place preference (also reported in mice by Donvito et al., 2019), nor did it modify reinstatement of a previously extinguished morphine conditioned place preference (Petrie et al., 2019). At the effective dose of 5 mg/kg, OlGly had no effects on its own in the absence of MWD. Although interference with the affective properties of MWD by OlGly was prevented by pretreatment with a CB_1_ receptor antagonist but not a PPARα antagonist (Petrie et al., 2019), interference with somatic MWD responses by OlGly was prevented by either pretreatment with a CB_1_ receptor antagonist or a PPARα antagonist (which had no effects on their own). Since OlGly does not strongly bind to CB_1_ receptors, the interference with the anti-withdrawal effects of OlGly on CB_1_ receptors was most likely mediated by its action as a FAAH inhibitor, and subsequent elevation of endogenous AEA levels.

Because endogenous OlGly is rapidly deactivated by amidases, a more stable analog, *N*-oleoylalanine (OlAla), was synthesized by Mechoulam’s group. OlAla, which is also an endogenous lipid (Bradshaw et al., 2009), also interfered with the affective symptoms of acute naloxone-precipitated MWD, but it was effective for a longer duration than OlGly through a CB_1_ receptor and PPARα -dependent mechanism (Ayoub et al., 2020). Accordingly, OlAla was found to inhibit FAAH and activate PPARα *in vitro*.

If OlGly and OlAla are effective in the treatment of MWD, it is important to determine if they might also modify tolerance to morphine at the most effective therapeutic dose of 5 mg/kg, ip, when tested under similar housing conditions as our previous work. Indeed, recent evidence indicates that the FAAH inhibitor, URB597, prevents tolerance to morphine nociception in the tail immersion test (Fotio et al., 2020). Here we evaluated the potential of OlGly and OlAla to modify tolerance to nociception, hyperthermia, and suppression of activity produced by morphine under a similar morphine treatment regime as employed by Fotio et al. (2020).

## Materials and Methods

### Animals

All animal procedures complied with the Canadian Council on Animal Care and were approved by the Institutional Animal Care Committee at the University of Guelph. Naïve male Sprague-Dawley rats (*n* = 64), weighing between 215 and 244 g on the day of habituation, obtained from Charles River Laboratories (St Constant, Quebec), were used. The rats were pair-housed in opaque plastic shoebox cages (48 × 26 × 20 cm), containing bed-o-cob bedding from Harlan Laboratories, Inc. (Mississauga, Ontario), a brown paper towel, and Crink-l′Nest^TM^ (The Andersons, Inc., Maumee, Ohio). Additionally, the rats were provided with a soft white paper container that was 14 cm long and 12 cm in diameter. The colony room was kept at an ambient temperature of 21°C with a 12/12 h light-dark schedule (lights off at 7 am). The rats were tested in their dark cycle and were maintained on chow and water *ad libitum*.

### Drugs

Morphine (MOR) was prepared in saline (SAL) at a concentration of 10 or 20 mg/ml and administered subcutaneously (sc) at 1 ml/kg (10 or 20 mg/kg, respectively). OlGly and OlAla were dissolved in a vehicle (VEH) mixture of ethanol, Tween 80, and physiological SAL in a 1:1:18 ratio. OlGly and OlAla were first dissolved in ethanol, Tween 80 was then added to the solution, and the ethanol was evaporated off with a nitrogen stream, after which, the SAL was added. The final VEH consisted of 1:9 (Tween80:saline). OlGly and OlAla were prepared at a concentration of 5 mg/ml and was administered intraperitoneally (ip; 1 ml/kg).

### Apparatus

#### Locomotor Test

To assess locomotor activity, a locomotor chamber made of black Plexiglas (60 × 25 × 25 cm) was placed in a room illuminated by a red light. The locomotor activity of each rat was captured by a video camera placed above the chamber and sent to the Ethovision software program (Noldus, Inc., NL) to measure distance (cm) traveled.

#### Tail Flick Test of Nociception

For the tail flick test, a water bath was maintained at 50°C. The rats were lightly restrained, and the distal two-thirds of the tail was submerged in the water bath. The time (sec) the rat kept its tail in the water was recorded. Two withdrawal response latencies were taken, 1 min apart to obtain an average tail flick latency. To protect against tissue injury, the test was stopped after 10 s if the animal did not flick its tail.

#### Body Temperature

Body temperature measures were taken using an infrared digital ear thermometer (Model: KI-8170, Life Brand; Shoppers Drug Mart Inc., Canada). Rats were lightly restrained, and the probe tip of the thermometer was carefully inserted into the top of the ear canal and held in place until a reading was obtained. Three consecutive body temperature measures were taken to obtain an average body temperature measure.

### Procedures

The experimental schedule is presented in Table 1. Following arrival in the facility, to establish baseline, all rats underwent the locomotor activity test, tail flick test and body temperature measures. On the baseline day and subsequent testing days, rats were first placed in the locomotor activity chambers for 15 min. They were then tested in the tail flick test, followed by body temperature measures as depicted in Table 1. Rats were randomly assigned to receive an injection of OlGly (5 mg/kg, ip, Experiment 1), OlAla (5 mg/kg, ip, Experiment 2) or VEH 10 min prior to an injection of SAL or morphine (10 mg/kg on Day 01 and 20 mg/kg for all subsequent days, sc) injections twice daily (beginning at 8 AM and 4 PM) for 13 days. This resulted in the following groups for Experiment 1 (*n* = 8/group): VEH-SAL, VEH-MOR, OlGly-SAL, OlGly-MOR, and the following groups for Experiment 2 (*n* = 8/group): VEH-SAL, VEH-MOR, OlAla-SAL, OlAla-MOR.

**TABLE 1:** Experimental schedule.

Day	Morning Injection OlGly or OlAla Beginning at 7:50 AM MOR or SAL Beginning at 8 AM	Locomotion Test Beginning at 8:30 AM 15 min	Tail Flick Test Beginning at 8:50 AM	Temperature Beginning at 9 AM	Afternoon Injections OlGly or OlAla Beginning at 3:50 PM MOR or SAL Beginning at 4 PM	Morphine Dose (mg/kg, s)
Baseline		X	X	X		
Day 01	X	X	X	X	X	10
Day 02	X				X	20
Day 03	X		X	X	X	20
Day 04	X				X	20
Day 05	X	X	X	X	X	20
Day 06	X				X	20
Day 07	X		X	X	X	20
Day 08	X				X	20
Day 09	X	X	X	X	X	20
Day 10	X				X	20
Day 11	X		X	X	X	20
Day 12	X				X	20
Day 13	X	X	X	X	X	20

### Statistical Analysis

For Experiments 1 and 2, the distance (cm) traveled in the activity test of each rat were entered into a 2 × 2 × 5 mixed factors ANOVA with the factors of Chronic Treatment (SAL or MOR) × Pretreatment (VEH and OlGly or OlAla) × Day. The mean seconds to flick the tail from the two tests of nociception were entered into a 2 × 2 × 8 mixed factors ANOVA for Experiments 1 and 2. The mean body temperature of the three measures were entered into a 2 × 2 × 8 mixed factors ANOVA for Experiments 1 and 2.

## Results

### Experiment 1

Ability of OlGly to modify tolerance to nociception, hyperthermia, and suppression of locomotor activity produced by morphine.

**FIGURE 1:**
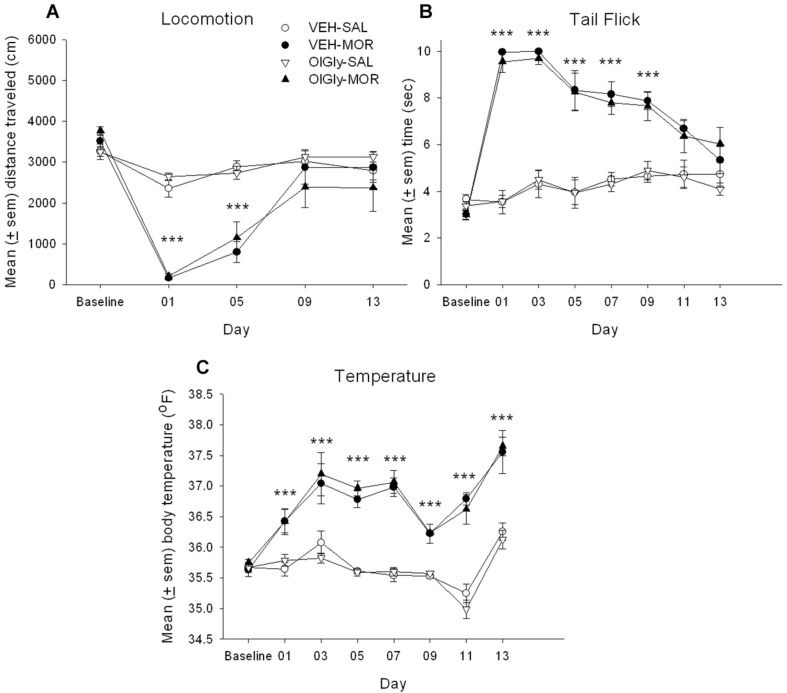
OlGly **(A)** the mean (± sem) distance (cm) traveled in the locomotion test, **(B)** the mean (± sem) latency to tail flick from a hot water bath, **(C)** mean (± sem) body temperature (°F), displayed by the various groups across the 13 days of chronic morphine. Asterisks indicate a significant difference from SAL, ***p < 0.001.

Rats developed tolerance to the hypolocomotor effects and to the analgesic, but not to the hyperthermic, effects of morphine. However, OlGly did not interfere with the establishment of tolerance. Figure 1A presents the mean (± sem) distance (cm) traveled in the locomotion test by the various groups across the 13 days of chronic morphine. The mixed factors ANOVA for activity revealed a significant effect of chronic treatment by day, *F*(4, 112) = 25.4; *p* < 0.001. On Days 01 and 05, rats administered morphine were less active than those administered saline (*p*’s < 0.001), but not on Days 09, 13 or baseline. Figure 1B presents the mean (± sem) latency to tail flick from a hot water bath for the groups across the 13 days of chronic morphine. The mixed factors ANOVA for tail flick latency revealed a significant effect of chronic treatment by day, *F*(7, 196) = 29.2; *p* < 0.001. On Days 01–09, rats administered chronic morphine showed longer tail flick withdrawal latencies than those administered chronic saline (*p*’s < 0.001), but they did not differ at baseline or Days 11–13. Figure 1C presents the mean (± sem) body temperature (°F) displayed by the various groups across the 13 days of morphine administration. The mixed factors ANOVA for body temperature revealed a significant chronic drug by day effect, *F*(7, 196) = 14.0; *p* < 0.001. Across all days (except for baseline), rats administered chronic morphine had higher body temperatures than rats administered saline (*p*’s < 0.001). OlGly did not modify any behavioral effect of morphine across the 13 days of chronic morphine exposure.

### Experiment 2

Ability of OlAla to modify tolerance to nociception, hyperthermia, and suppression of locomotor activity produced by morphine.

**FIGURE 2:**
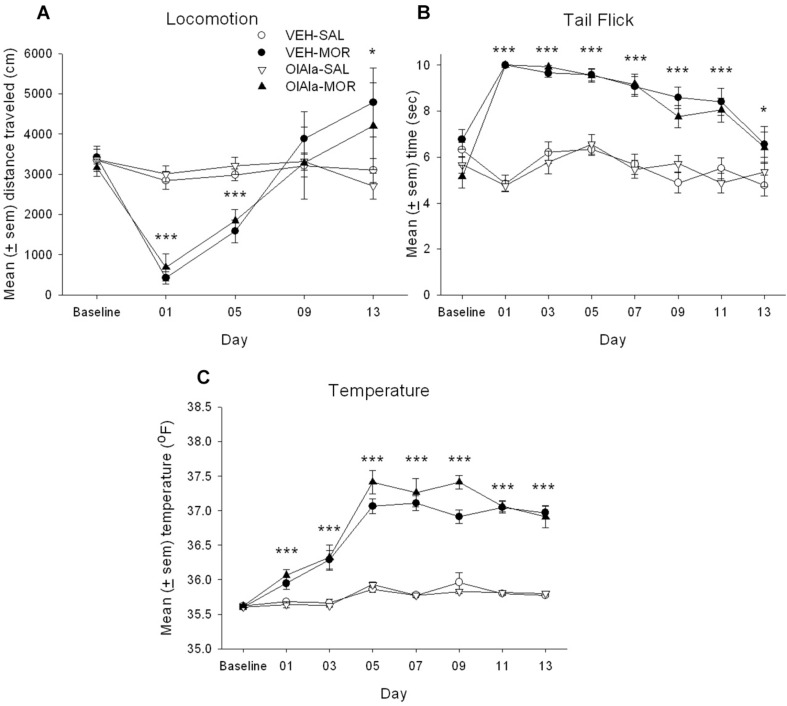
OlAla **(A)** the mean (± sem) distance (cm) traveled in the locomotion test, **(B)** the mean (± sem) latency to tail flick from a hot water bath, **(C)** mean (± sem) body temperature (°F), displayed by the various groups across the 13 days of chronic morphine. Asterisks indicate a significant difference from SAL, ***p < 0.001, *p < 0.05.

Much like in Experiment 1, rats developed tolerance to the hypolocomotor effects and to the analgesic, but not to the hyperthermic, effects of morphine. However, OlAla did not interfere with the establishment of tolerance. Figure 2A presents the mean (± sem) distance (cm) traveled in the locomotion test by the various groups across the 13 days of chronic morphine. The mixed factors ANOVA for activity revealed a significant effect of chronic treatment by day *F*(4, 112) = 14.6; *p* < 0.001. On Days 01, 05 (*p*’s < 0.001), and 13 (*p* < 0.05), rats administered chronic morphine were less active than those administered chronic saline, but not at baseline. Figure 2B presents the mean (± sem) latency to tail flick for the groups across the 13 days of chronic morphine. The mixed factors ANOVA for tail flick revealed a significant effect of chronic treatment by day *F*(7, 196) = 18.6; *p* < 0.001. On Day 01–11 (*p*’s < 0.001), and 13 (*p* < 0.05), rats administered chronic morphine showed longer tail flick withdrawal latencies than those administered chronic saline, but they did not differ at baseline. Figure 2C presents the mean (± sem) body temperature (°F) displayed by the various groups across the 13 days of morphine administration. The mixed factors ANOVA for body temperature revealed a significant chronic drug by day effect, *F*(7, 196) = 41.9; *p* < 0.001. Across all days (except for baseline), rats administered chronic morphine had higher body temperatures than rats administered saline (*p*’s < 0.001). OlAla did not modify any behavioral effect of morphine across the 13 days of chronic morphine exposure.

## Discussion

Chronic dosing of morphine (twice daily 20 mg/kg, except day 1, for 13 days) resulted in tolerance to the hypolocomotor and the anti-nociceptive, but not to the hyperthermic, effects of morphine, as has been reported to the anti-nociceptive effects of morphine by Fotio et al. (2020). Here rats were injected with OlGly (5 mg/kg, ip, Experiment 1) or OlAla (5 mg/kg ip, Experiment 2) prior to each chronic treatment with morphine or saline to determine if these fatty acid amides that act *in vitro* as FAAH inhibitors and PPARα agonists (Donvito et al., 2019; Ayoub et al., 2020), would interfere with morphine tolerance as has been reported for the FAAH inhibitor, URB597 (Fotio et al., 2020). However, at least at a dose of 5 mg/kg, ip, neither OlGly nor OlAla administration interfered with the establishment of tolerance to the anti-nocicieptive and the hypolocomotor effects of morphine. These results suggest that, at the most effective dose for interference with acute naloxone-precipitated MWD responses (Petrie et al., 2019; Ayoub et al., 2020; Rock et al., 2020), neither OlGly nor OlAla are likely to prevent the establishment of tolerance to the chronic effects of morphine. However, it is possible that a higher dose of OlGly or OlAla would be required to chronically reduce tolerance than that required to acutely reduce opioid withdrawal. It is also possible that the doses of OlGly or OlAla employed here would interfere with tolerance to a lower dose of chronically administered morphine. As well, as this work has been conducted only with male rats, it is conceivable that female rats may respond differently to the effects of these fatty acid amides on tolerance to morphine or on naloxone-precipitated MWD effects.

Unlike OlGly and OlAla, the FAAH inhibitor URB597, has recently been shown to prevent tolerance to morphine anti-nociception in the tail flick test using a similar procedure and dosing schedule as was used here (Fotio et al., 2020). The effect of URB597 on morphine tolerance was reversed by pretreatment with a cannabinoid 2 (CB_2_) receptor antagonist (AM630) and partially suppressed by pretreatment with a CB_1_ receptor antagonist (AM251) or the PPARα antagonist (GW6471). Additionally, AEA mobilization and the mRNA levels of the PPARα receptor were elevated in the spinal cord of morphine tolerant mice (Fotio et al., 2020). Since OlGly and OlAla interfere with naloxone-precipitated MWD by both CB_1_ and PPARα antagonism (Ayoub et al., 2020; Rock et al., 2020), we predicted that OlGly and OlAla would also interfere with tolerance to morphine as does URB597 (Fotio et al., 2020). The lack of an effect on tolerance to morphine may have been a function of the relatively weak efficacy of OlGly to inhibit FAAH (IC_50_ = 8.65 μM; Donvito et al., 2019) relative to the more potent FAAH inhibitor, URB597 (4.6 nM; Mor et al., 2004). Similar to OlGly, OlAla weakly inhibited FAAH by about 40% at 10 μM (Ayoub et al., 2020). Unfortunately, neither FAAH activity nor AEA levels were measured in the tissue of these animals. Thus, it is possible that, at the dose used, neither compound elevated the brain concentrations of AEA and other *N*-acylethanolamines to an extent sufficient to activate CB1 or PPARa, and that a higher dose of OlGly and/or OlAla may have been more effective in inhibiting FAAH and may therefore have modified tolerance to morphine.

We have previously demonstrated that at a dose of 5 mg/kg, OlGly and OlAla interfere with naloxone-precipitated withdrawal from acutely administered morphine, without modifying morphine reward (Donvito et al., 2019; Petrie et al., 2019; Ayoub et al., 2020; Rock et al., 2020). Here we show that this effective dose of OlGly and OlAla did not affect the hypolocomotor effect, anti-nociceptive effect or the hyperthermic effect of morphine on any occasion across 13 treatment days and did not modify the development of tolerance to any of these effects. It is conceivable that either higher doses of the fatty acid amides or a different treatment regime of morphine may have revealed an effect of OlGly or OlAla on tolerance to one or more effects of morphine. As the work to date with OlGly and OlAla has been limited to acute naloxone precipitated MWD, future studies will determine the potential of OlGly to interfere with withdrawal reactions (spontaneous and naloxone-precipitated) from chronic treatment with opiates. However, on the basis of the acute MWD effects, it is unlikely that treatment with OlGly or OlAla to reduce MWD will reduce the potential of morphine to produce analgesia in clinical populations. These results suggest that these fatty acid amides may be used effectively in the future to combat MWD without compromising the beneficial therapeutic effects of opiates.

## Data Availability Statement

The raw data supporting the conclusions of this article will be made available by the authors, without undue reservation.

## Ethics Statement

The animal study was reviewed and approved by the University of Guelph Animal Care Committee.

## Author Contributions

ER, CL, and MS performed the behavioral testing with assistance in daily injections from MD. LP, and ER wrote the manuscript, with suggestions and revisions from CL. LP, CL, and ER conceived and coordinated the work. All authors read, contributed to, and approved the final version of the manuscript.

## Conflict of Interest

The research was supported in part by funding from Plant Ext who hold a license on the patent for OlGly and OlAla as treatments for addiction. The authors declare that the research was conducted in the absence of any commercial or financial relationships that could be construed as a potential conflict of interest.
